# Adverse effect of assisted reproductive technology-related hyperoestrogensim on the secretion and absorption of uterine fluid in superovulating mice during the peri-implantation period

**DOI:** 10.3389/fendo.2023.859204

**Published:** 2023-03-06

**Authors:** Xinru Xia, Yuan Zhang, Meng Cao, Xiang Yu, Li Gao, Lianju Qin, Wei Wu, Yugui Cui, Jiayin Liu

**Affiliations:** ^1^ State Key Laboratory of Reproductive Medicine, Center for Clinical Reproductive Medicine, First Affiliated Hospital, Nanjing Medical University, Nanjing, China; ^2^ Department of Pediatrics, First Affiliated Hospital, Nanjing Medical University, Nanjing, China

**Keywords:** assisted reproductive technology, hyperoestrogenism, embryo implantation, uterine fluid, superovulation

## Abstract

**Objectives:**

This study aimed to investigate the potential mechanism of hyperoestrogensim elicited by ovulation induction affects endometrial receptivity and leads to embryo implantation abnormality or failure.

**Study design:**

Establishment of ovulation induction mouse model. Changes in mouse body weight, ovarian weight, serum E2 level and oestrous cycle were observed. During the peri-implantation period, morphological changes in the mouse uterus and implantation sites and the localization and protein levels of oestrogen receptors ERα and ERβ, the tight junction factors CLDN3 and OCLN, the aquaporins AQP3, AQP4 and AQP8, and the sodium channel proteins SCNN1α, SCNN1β and SCNN1**γ** were observed. The expression and cellular localization of ERα, CLDN3, AQP8 and SCNN1 β in RL95-2 cell line were also detected by western blotting and immunofluorescence.

**Results:**

Ovarian and body weights were significantly higher in the 5 IU and 10 IU groups than in the CON group. The E2 level was significantly higher in the 10 IU group than in the CON group. The mice in the 10 IU group had a disordered oestrous cycle and were in oestrus for a long time. At 5.5 dpc, significantly fewer implantation sites were observed in the 10 IU group than in the CON (p<0.001) and 5 IU (p<0.05) groups. The probability of abnormal implantation and abortion was higher in the 10 IU group than in the CON and 5 IU groups. CLDN3, OCLN, AQP8 and SCNN1β in the mouse endometrium were localized on the luminal epithelium and glandular epithelium and expression levels were lower in the 10 IU group than in the CON group. The protein expression level of ERα was increased by 50% in the 10 IU group compared to the CON group. The expressions of CLDN3, AQP8, SCNN1β in RL95-2 cell line were significantly depressed by the superphysiological E2, ERα agonist or ERβ agonist, which could be reversed by the oestrogen receptor antagonist.

**Conclusion:**

ART-induced hyperoestrogenism reduces CLDN3, AQP8 and SCNN1β expression through ERα, thereby destroying tight junctions and water and sodium channels in the endometrial cavity epithelium, which may cause abnormal implantation due to abnormal uterine fluid secretion and absorption.

## 1 Introduction

Although assisted reproductive technology (ART) has been significantly improved and overcomes many potential causes of infertility, the pregnancy success rate is still relatively low, mainly due to the failure of embryo implantation (1–6).

Embryo implantation, the most critical step in mammalian pregnancy (7), is an extremely complex physiological process regulated by a variety of factors, and its underlying mechanism has not yet been fully elucidated (7, 8). It requires an implantable blastocyst and a receptive endometrium, which communicate and interact with each other to achieve conception (8, 9). Oestrogen and progesterone are the main hormones that regulate this process (6, 10–12). During ART, a large number of eggs need to be collected and fertilized to increase the number of high-quality embryos available for transfer. The development of multiple follicles is induced by hormone stimulation. Although this method can be used to select high-quality embryos for transfer, ovarian stimulation can also lead to superphysiological levels of E2 (13, 14). It has been reported that hyperoestrogensim during the fresh embryo transfer cycle of ovulation induction leads to a decrease in the embryo implantation rate and clinical pregnancy rate (15–22).

The main function of the endometrium, the main target of oestrogen, is embryo implantation. Oestrogen mainly acts through two classic oestrogen receptor subtypes, ERα and ERβ, in the endoderm. Studies have revealed that ERα knockout mice are infertile, while embryo implantation does not seem to be disturbed in ERβ knockout mice (23). Elevated levels of sex steroids may impair endometrial receptivity (5, 21, 22, 24), leading to decreases in the embryo implantation and clinical pregnancy rates. However, the mechanism underlying the effect of superphysiological E2 levels on embryo implantation is still unclear.

Tight junctions (TJs) exist between the epithelial or endothelial cells of vertebrates that function to tightly join the plasma membranes of adjacent cells, and no gaps are present in cell junctions (25). TJs mainly function to close the gaps between adjacent cells, prevent molecules in solution from penetrating the body through gaps between cells, and maintain the relative stability of the bodily environment (26). TJs are composed mainly of members of two protein superfamilies, namely, the transmembrane protein family, which includes occludin (OCLN) and CLDN, and the perimembrane protein family, which includes zonula occludens (ZO) (27). Whether ovarian stimulation alters the structure and function of TJs among endometrial epithelial cells by changing the expression of TJ proteins, leading to abnormal embryo implantation, has not been reported.

Aquaporins in the mammalian reproductive system mainly function to regulate the amount of water in the uterus and fallopian tubes (28–32). The latest research shows that the expression of Aqp3, Aqp4, Aqp5 and Aqp8 is induced by E2 but not P4, while the expression of Aqp1 and Aqp11 is increased by P4. P4 inhibits the expression of Aqp3 and Aqp4 induced by E2, and E2 inhibits the expression of Aqp1 and Aqp11 induced by P4. Aqp9 expression is not significantly altered. Ovarian stimulation is known to alter the expression of D4 Aqp3, Aqp5 and Aqp8 (33). However, whether ovarian stimulation changes the amount of water in the uterine cavity by changing the expression of aquaporins and then disrupting embryo implantation has not been addressed.

Ion channels have proven to be essential for reproduction. An increasing number of studies have shown that ion channels in the endometrium play an important role in regulating endometrial receptivity and embryo implantation. Abnormal expression or function of endometrial ion channels may lead to impaired endometrial receptivity and/or implantation failure. The epithelial sodium ion channel (ENaC), which is encoded by the SCNN1 gene of the ENaC superfamily, is highly expressed in epithelial cells of the lung, kidney, brain, and reproductive tract. In the female reproductive tract, ENaC regulates the absorption of uterine fluid during the reproductive cycle. Thus far, the α, β, γ, and δ subunits of mammalian ENaC have been cloned. However, which ENaC subunits play important roles in ovarian stimulation has not been determined.

Therefore, this research focuses on the specific mechanism by which hyperoestrogensim regulates embryo implantation during ART-induced ovulation induction. It is technically and ethically difficult to study the process of human embryo implantation *in vivo*. Therefore, we established a hyperoestrogenic mouse model and observed whether it sufficiently simulates the clinical ovulation stimulation cycle of the embryo implantation process. We also used cell line RL95-2 to simulate the process of human embryo implantation *in vitro*. To investigate whether high oestrogen levels affect the mouse endometrium during the peri-implantation period and thus cause embryo implantation anomalies or failures and explored the possible mechanisms.

## 2 Materials and methods

### 2.1 Animals

All procedures involving the use of animals were approved by the Experimental Animal Ethics Committee of the University of Nanjing Medical University (Nanjing, China) with the ethics number IACUC-1702002.

Eight-week-old specific pathogen-free (SPF) ICR mice were bred under controlled environmental conditions (12 h light/dark cycle, relative humidity of 40–70%, and temperature of 20–25°C).

### 2.2 Treatment with gonadotropin to induce superovulation

The oestrous cycle phase was determined based on vaginal smears and staining with methylene blue (Shanghai Yuanye Biotechnology Co., Ltd., CAS: 7220-79-3). The oestrous cycle was divided into proestrus, oestrus, metestrus and dioestrus.

Female mice in proestrus or oestrus were injected intraperitoneally (ip) with 10 IU pregnant mare serum gonadotropin (PMSG, Sigma, USA) and then with 10 IU human chorionic gonadotropin (HCG, Sigma) 48 h later (the 10 IU group). Female mice in metestrus were injected ip with 5 IU PMSG and then with 5 IU HCG 48 h later (the 5 IU group).

Female mice in dioestrus were injected ip with physiological saline and then with physiological saline 48 h later (the CON group). Immediately after the HCG or physiological saline injection, female mice were mated with males at a ratio of 2:1. Mating was confirmed the next morning by the presence of a vaginal plug, and this day was considered 0.5 days postcoitum (dpc). If no vaginal plug was observed, vaginal smears were performed for one week.

We have 6 mice in each group. The mice were weighed at 8 a.m. At 3.5, 4.5 and 5.5 dpc, 0.7–0.8 ml of peripheral blood was taken from the inner canthal vein after anaesthetization with 0.2 ml/10 g tribromoethanol. Female mice were sacrificed after the injection of 0.1 ml/10 g trypan blue solution (Sigma, catalogue number: 93595) into their tail vein, and successful injection was confirmed by the mouths and ears of the mice turning blue. The uterus and ovaries were removed immediately and rinsed in precooled phosphate-buffered saline (PBS) (Beyotime, China, catalogue number: ST476). The uterus and ovaries were harvested and photographed immediately and then used for subsequent experiments. The number of implantation sites was determined by trypan blue staining. The ovaries were weighed; half of the uterus was fixed in 4% paraformaldehyde solution, and the other half was placed in a cryotube and stored at -80°C. The blood taken from the inner canthal vein was centrifuged at 3500 rpm for 5 min, and the supernatant was then stored at -80°C before the analysis of 17β-oestradiol levels. The next morning, the 4% paraformaldehyde was replaced with 75% ethanol, and 5 μm sections were used for haematoxylin and eosin (H&E) staining and immunohistochemistry (IHC).

The body weight, size and weight of the ovaries, serum E2 level, oestrous cycle, size and number of implantation sites in the uterus, and localization and expression of oestrogen receptors, TJ proteins, aquaporins and sodium channel proteins in endometrial epithelial cells were assessed.

### 2.3 Cells and cell culture techniques

RL95-2 cells (an endometrial adeno-carcinoma cell line with microvilli on the cell surface) were maintained in 25-cm^2^ flasks using Dulbecco’s minimal essential medium (DMEM)/F12 supplemented with 10% (vol/vol) fetal bovine serum (FBS). The cells were maintained at 37°C in a humidified atmosphere and 5% CO2. RL95-2 cells were initially passaged using a standard trypsinization protocol, plated in 24-well culture dishes, and grown to 70% confluence. The cells were then grown in serum-free, phenol red-free medium for 12 hours before the experimental treatments. The cells were then treated for another 24 hours with either 10^-6^M E2 (Sigma, USA, catalogue number: E8875), 10^-6^M ERα agonist (PPT) (Tocris, UK, catalogue number: 1026), 10^-6^M ERβ agonist (DPN) (Tocris, UK, catalogue number: 1494), or 10^-6^M ER antagonist (ICI 182,780) (Tocris, UK, catalogue number: 1047).

### 2.4 Analysis of serum 17β-oestradiol levels

A chemiluminescence detection kit (oestradiol determination kit, Beckman Coulter) and a chemiluminescence instrument (UniCel DxI 800, Beckman Coulter) were used for this analysis. Both the intra- and interassay variation were within the set range.

### 2.5 H&E staining

The morphological features of the implantation site were analysed by H&E staining. Uterine tissues isolated from mice were fixed with 4% paraformaldehyde overnight, embedded in paraffin, and cut into 5-μm-thick sections. The sections were deparaffinized and hydrated by brief incubations in xylene, ethanol, and water. Then, the sections were stained with haematoxylin, rinsed, and stained with eosin. The stained sections were dehydrated by brief incubations in water, alcohol and xylene. After mounting, the sections were observed with a bright-field microscope.

### 2.6 IHC

The expression of oestrogen receptors, TJ proteins, aquaporins and sodium channel proteins was assessed by IHC. The primary antibodies utilized are listed in **Table 1**. PBS rather than the primary antibody served as the negative control.

**Table 1:** Primary antibodies used.

Primary antibody	Host	Company	Catalogue number	Dilution (IHC)	Dilution (WB)
**ERα**	Rabbit	abcam	ab32063	1:200	1:1000
**ERβ**	Mouse	santa	sc-390243	1:100	1:500
**CLDN3**	Rabbit	abcam	ab52231	1:100	1:500
**OCLN**	Mouse	santa	sc-133256	1:200	1:1000
**AQP3**	Rabbit	abcam	ab125219	1:100	1:500
**AQP4**	Rabbit	abcam	ab259318	1:100	1:500
**AQP8**	Mouse	santa	sc-81870	1:100	1:500
**SCNN1α**	Rabbit	sigma	SAB5200105	1:100	1:500
**SCNN1β**	Rabbit	sigma	SAB5200106	1:100	1:500
**SCNN1γ**	Rabbit	sigma	SAB5200107	1:100	1:500

IHC, immunohistochemistry; WB, Western blot

Tissue was fixed overnight in 4% paraformaldehyde, embedded in paraffin, and cut into 5-μm-thick sections. The sections were deparaffinized, hydrated, and incubated in a histochemistry box containing 10 mM citrate buffer (pH 6.0) at 95°C for 15 min for antigen retrieval. The sections were pretreated with 3% H2O2 in 0.1 M Tris-buffered saline (TBS, pH 7.4) for 10 min to block endogenous peroxidase activity. The sections were washed with PBS three times for 3 min each and then treated with protein blocking solution at 37°C for 10 min and with a primary antibody (diluted 1:100 in 5% BSA or IHC primary antibody dilution buffer) overnight at 4°C. The sections were washed with PBS three times for 3 min each time, covered with enzyme-labelled anti-mouse/rabbit polymer or Solution C and incubated for 10 min at room temperature. They were then covered with horseradish peroxidase (HRP)-labelled streptavidin or Solution D and incubated at room temperature for 10 min. The slides were washed thoroughly with PBS (pH=7.4) between incubations.

After treatment with 3,3’-diaminobenzidine (DAB) chromogen substrate solution, peroxidase bound to the antibody complex was observed. The DAB reaction was monitored under a microscope to determine the optimal incubation time, and the reaction was stopped by washing with 0.1 M TBS multiple times. The immunolabelled sections were dehydrated *via* a graded ethanol series, cleared in xylene, and fixed. The slides were counterstained with haematoxylin before mounting. Brown deposits indicated positive signals and were evaluated under a Nikon Eclipse Ti microscope (Nikon, Japan).

### 2.7 Immunofluorescence analysis

RL95-2 cells were fixed in 4% paraformaldehyde for 20min. Sections were washed three times with phosphate buffered saline (PBS) for 5 min. Treat with 0.4% Triton X-100 for 10min, and then washed three times with PBS for 5min. Non-specific binding was blocked with 5% bovine serum albumin (BSA) for 30min. Sections were incubated with the following primary antibodies diluted (1:100) in blocking solution (5% BSA) overnight at 4°C. ERα (Abcam ab32063), CLDN3 (Invitrogen, 34-1700), AQP8 (bioworld, BS71279) and SCNN1β (Sigma, SAB5200106). Sections were then washed three times with PBS for 5 min. For the fluorescent detection Alexa Fluor™ 488 goat anti-rabbit (dilution 1:500, Thermo Fisher Scientific) secondary antibody was used and nuclear counterstaining was performed with 4,6-diamidino-2-phenylindole (DAPI, Life Technologies, 10236276001). Evaluation of the sections was performed using confocal microscopy (Nikon, Eclipse Ti, Japan).

### 2.8 Western blot analysis

Sodium dodecyl sulfate-polyacrylamide gel electrophoresis (SDS-PAGE) was performed routinely. Frozen uterine samples were homogenized in tissue lysis buffer (50 mM Tris-HCl pH 7.5, 100 mM NaCl, 2 mM EDTA, 0.1% SDS, 1% NP40) containing 1% protease and phosphatase inhibitors. The tissue homogenates were clarified by centrifugation at 12,000×g for 15 min at 4°C. A BCA protein concentration determination kit (Biyuntian, China, catalogue number: P0010) was used to determine the protein concentration, and the clarified supernatants were mixed with 6× SDS loading buffer (5:1) and transferred to a 70°C water bath for 10 min to denature the proteins. The samples were separated on NuPAGE™ 7% Tris-acetate protein gels (Invitrogen, catalogue number: EA0358BOX) and electrotransferred onto a PVDF Hybond nitrocellulose membrane (Millipore). The membrane was blocked in TBST (10 mM Tris-HCl (pH 7.5), 150 mM NaCl, and 0.05% (w/v) Tween 20) containing 5% skim milk for 1 h at room temperature. Then, the membrane was incubated with a primary antibody (1:1000 dilution) at 4°C overnight, and a rabbit anti-mouse GAPDH polyclonal antibody (1:5000 dilution, Bioworld, catalogue number: AP0063) was used as a control. After washing with TBST, the membrane was incubated in 5% skim milk in TBST containing a secondary antibody (diluted 1:5000, HRP-conjugated goat anti-rabbit IgG, Invitrogen, catalogue number: Ab6721; HRP-conjugated goat anti-mouse IgG), Invitrogen, catalogue number: Ab6789) for one hour at room temperature. The membrane was washed 3 times with TBST, and an ECL kit (Thermo, catalogue number: 1863096; 1863097) was then used to visualize the bands. The membrane was scanned with a luminescence imaging analyser. The relative protein levels were evaluated with ImageJ analysis software (National Institutes of Health, Maryland, Baltimore, USA). The concentration in each sample is expressed as the grey value relative to that of GAPDH or ACTIN.

### 2.9 Statistical analysis

The data are presented as the means ± SDs of three or more independent experiments. For group comparisons, one-way ANOVA or Student’s *t* test was performed using Prism software version 5.0 for statistical data analysis (GraphPad Software, Inc.). Differences were considered significant at *p < 0.05, **p< 0.01, and ***p< 0.001.

## 3 Results

### 3.1 Characteristics of female mice after PMSG/HCG injection

To investigate the effects of superovulation treatments on female ​mice, 5 IU or 10 IU PMSG/HCG was injected ip into the mice. We assessed their body weight, ovarian morphology, ovarian weight, serum oestradiol levels and oestrous cycle. There was no significant difference in the mouse weight, which was measured every other day, among the different groups (**Supplemental Figure 1**).

### 3.2 Ovarian morphology and ovarian weight of the CON, 5 IU and 10 IU groups from 3.5 to 5.5 dpc

**Figure 1:**
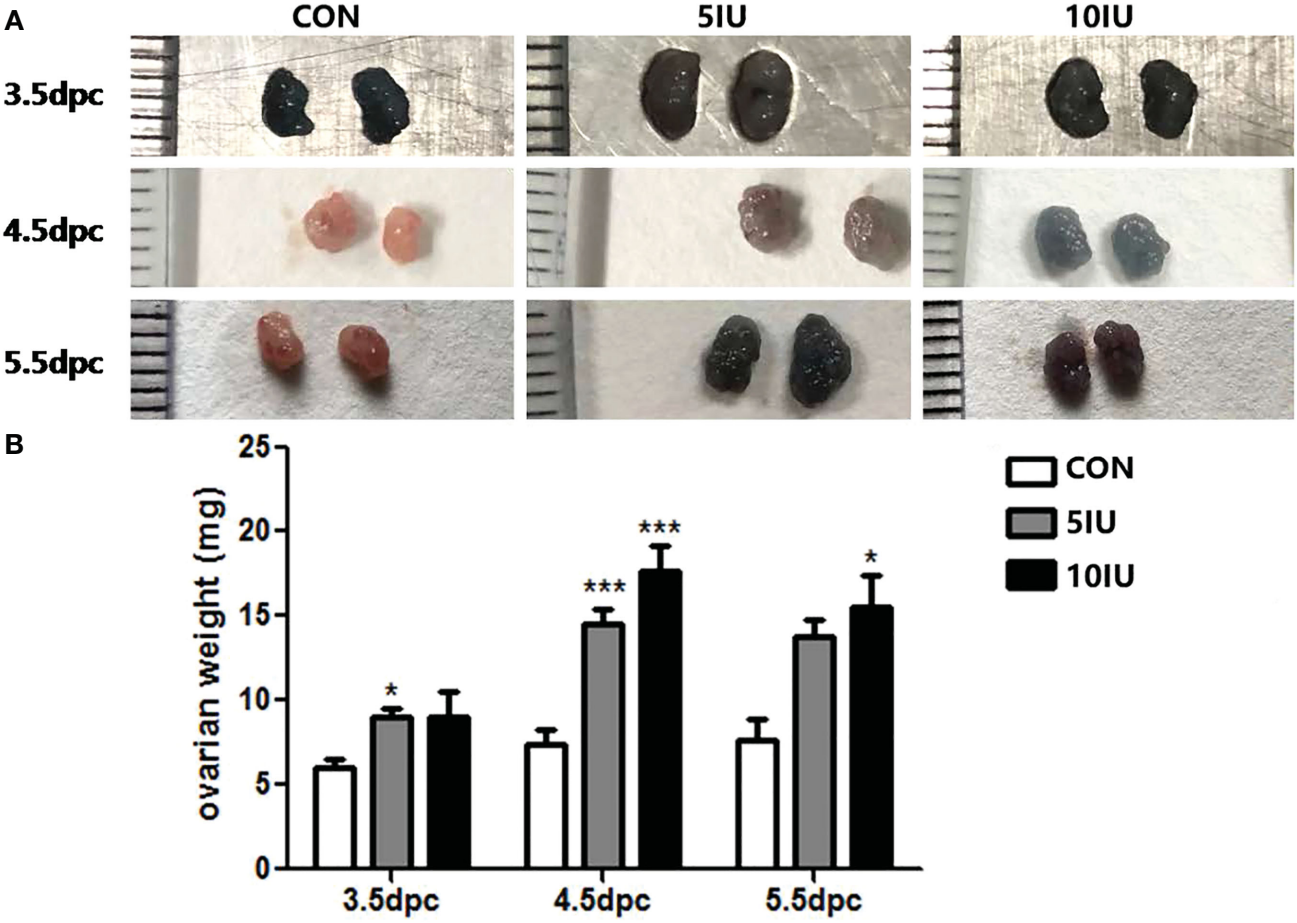
Ovarian morphologies and ovarian weights in the CON, 5 IU and 10 IU groups from 3.5 dpc to 5.5 dpc. **(A)** Changes in ovarian morphology from 3.5-5.5 dpc among the three groups. **(B)** Change in ovarian weight from 3.5-5.5 dpc among the three groups. The blue colour of the ovary in Figure A is due to the injection of trypan blue into the tail vein of the mouse. Compared with the control group, *p<0.05, ***p<0.001.

During the peri-implantation period (from 3.5 to 5.5 dpc), the ovaries of the mice in the 5 IU and 10 IU groups were larger than those of the mice in the CON group (**Figure 1A**), and the ovarian weight showed a dose-dependent increase, as the ovarian weights of the 5 IU and 10 IU groups were increased compared with that of the CON group (**Figure 1B**). However, there was no significant increase in the mouse weight (**Supplemental Figure 1**). These changes were consistent with the clinical characteristics of ovaries after superovulation.

### 3.3 Differences in the serum oestrogen levels and oestrous cycles between the CON and 10 IU groups

**Figure 2:**
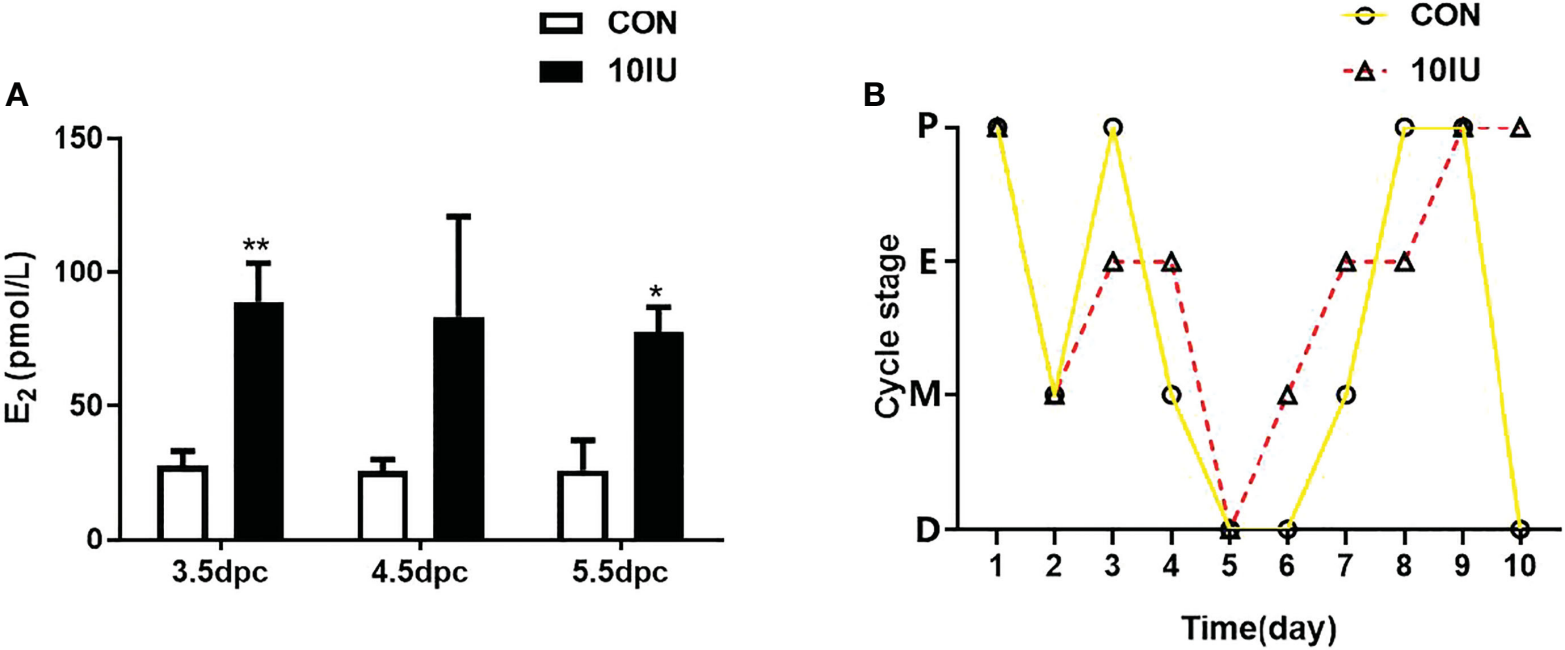
Serum oestrogen level and oestrus cycle changes between the CON and 10 IU groups. **(A)** Serum oestrogen levels of mice in the CON and 10 IU groups during the peri-implantation period. **(B)** Changes in the oestrous cycle of the mice in the CON and 10 IU groups. The abscissa depicts the number of days of detection, and the ordinate depicts the oestrus cycle. P: pro-oestrus, E: oestrus, M: meta-oestrus, D: dioestrus. Compared with the control group, *p<0.05, **p<0.01.

We measured the peripheral serum oestradiol and progesterone concentrations during the peri-implantation period. The serum oestradiol level in the 10 IU group was 2-fold higher than that in the CON group (**Figure 2A**), while the serum progesterone level in the 10 IU group increased by 100% at 3.5dpc and increased by 50% at 4.5dpc compared to the CON group, but the difference was not statistically significant (**Supplemental Figure 2**). The mice in the CON group had a normal oestrus cycle, progressing from proestrus to oestrus, metestrus and dioestrus. In contrast, the oestrous cycle of the 10 IU group was disordered, and these mice remained in oestrus for a long time (**Figure 2B**). The above results indicate that our model sufficiently simulates the endocrine hormone alterations caused by clinical superovulation and a long-term hyperoestrogen state.

### 3.4 Effect of superovulation on embryo implantation during the peri-implantation period

To study the effect of superovulation treatment on embryo implantation, we compared the uterine morphologies, implantation site numbers, and H&E staining of implantation sites among the three different groups.

### 3.5 Uterine morphologies and implantation site numbers in the CON, 5 IU and 10 IU groups during the peri-implantation period

**Figure 3:**
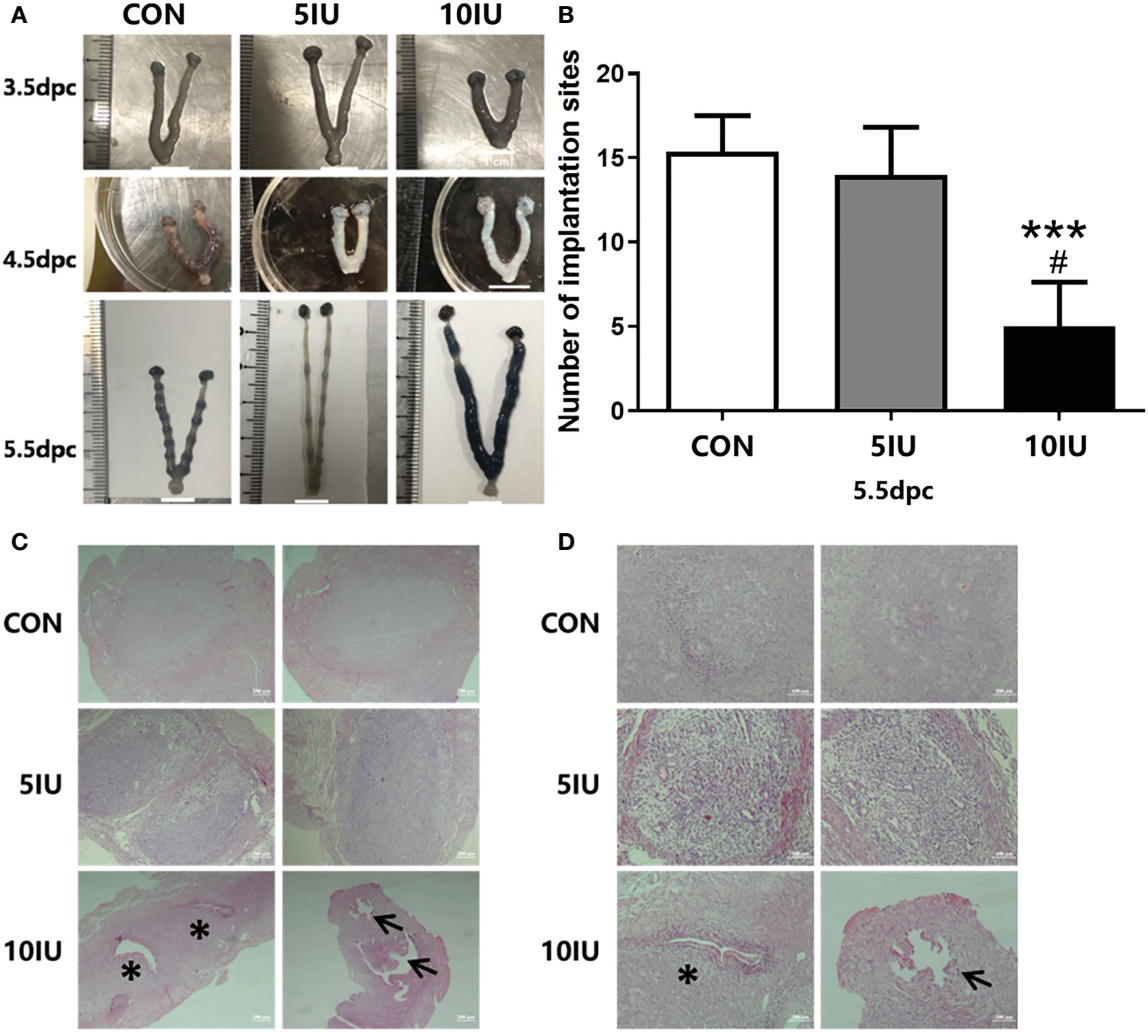
Uterine morphologies of mice during the peri-implantation period, implantation site numbers and implantation sites in the endometrium at 5.5 dpc among the CON, 5 IU and 10 IU groups. **(A)** Morphological changes in the mouse uteri from 3.5-5.5 dpc. A: Morphological changes in the uteri of mice from 3.5-5.5 dpc among the three groups. The implantation sites were stained with trypan blue. **(B)** Comparison of the implantation site numbers among the three groups of mice at of 5.5 dpc. Compared with the CON group, ***p<0.001. Compared with the 5IU group, ^#^p<0.05. **(C)** Implantation sites in the endometrium at 5.5 dpc among the CON, 5 IU and 10 IU groups. Bar=200 μm. **(D)** Implantation site in the endometrium at 5.5 dpc among the CON, 5 IU and 10 IU groups. Bar=100 μm. ***p<0.001, *p<0.05. The arrows indicate abnormal sites of embryo implantation. The asterisk indicates the site of miscarriage.

At 3.5 dpc, no embryos were implanted in the uteri of mice in the CON group, but the uteri of mice in the 10 IU group showed obvious oedema and stubs. At 4.5 dpc, the uteri of mice in the CON group began to show implantation sites, but those of mice in the 5 IU group and 10 IU group did not yet show obvious implantation sites. At 5.5 dpc, the uteri of mice in the CON group showed complete implantation, while implantation in the uteri of mice in the 5 IU group and the 10 IU group occurred later than that in the CON group, and the spacing between the implantation sites was uneven (**Figure 3A**). There were significantly fewer implantation sites in the 10 IU group than in the CON group (p<0.001) and the 5 IU group (p<0.05) (**Figure 3B**).

### 3.6 Endometrial implantation sites in the CON, 5 IU and 10 IU groups at 5.5 dpc

To investigate the effect of hyperoestrogenism on implantation sites in mice at 5.5 dpc, we performed H&E staining. Morphological changes in the uteri were observed. Embryos in the CON group were evenly spaced and of the same size, and no gap existed between the embryo and the inner membrane. The implantation sites were mainly normal, the embryo spacing was even, and the gap between the embryo and the inner membrane was not obvious in the 5 IU group compared with the CON group; however, the embryo sizes differed between the two groups. In the 10 IU group, the gap between the embryo and the inner membrane was large, the embryo spacing was uneven, and the embryos were too small, resulting in abnormal implantation (**Figure 3C**). The probability of entry and miscarriage was higher in the 10 IU group than in the CON and 5 IU groups (**Figure 3D**).

### 3.7 Possible mechanism by which hyperoestrogenism affects embryo implantation

To study the possible mechanism by which hyperoestrogenism disrupts embryo implantation during ovulation induction, IHC and western blotting were performed to assess the localization and expression of the oestrogen receptors, tight junction factors, the aquaporins and the sodium channel proteins in the endometria of 5.5 dpc mice and RL95-2cell line.

### 3.8 Localization and western blot analyses of the ERα and ERβ proteins in the mouse uterus during the peri-implantation period

**Figure 4:**
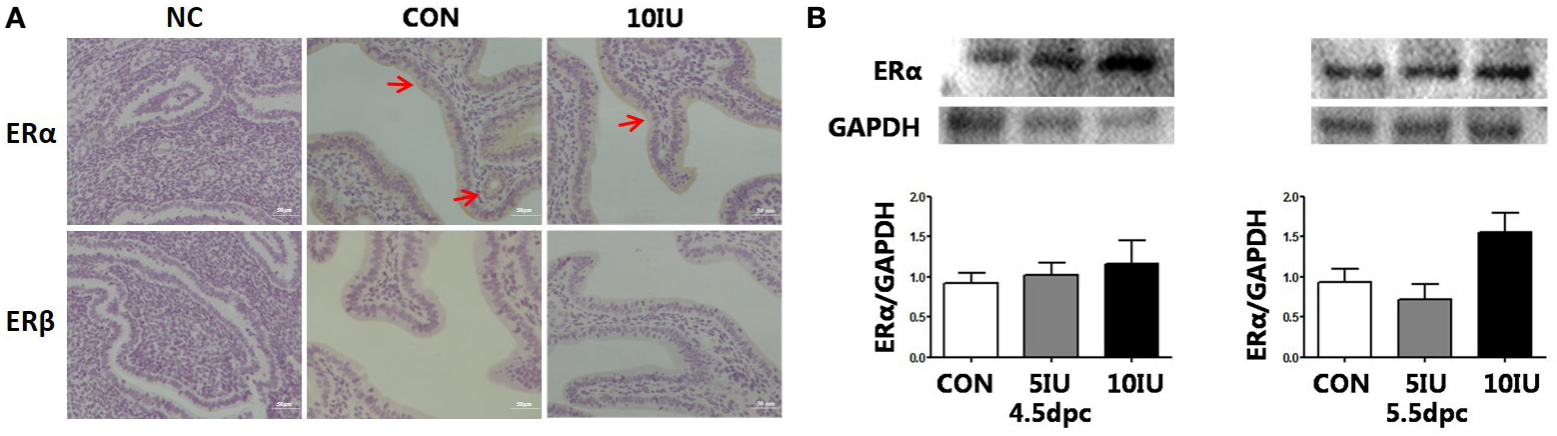
The localization and protein expression of the oestrogen receptors ERα and ERβ in the mouse endometrium at 4.5 dpc and 5.5 dpc in the CON, 5 IU and 10 IU groups. **(A)** Localization of ERα and ERβ in the endometria of mice at 5.5 dpc. Bar=50 μm. **(B)**: Protein expression of ERα in the endometria of mice in the CON, 5 IU and 10 IU groups at 4.5 dpc and 5.5 dpc. NC, negative control. The arrows indicate the localization of target protein.

**Figure 5:**
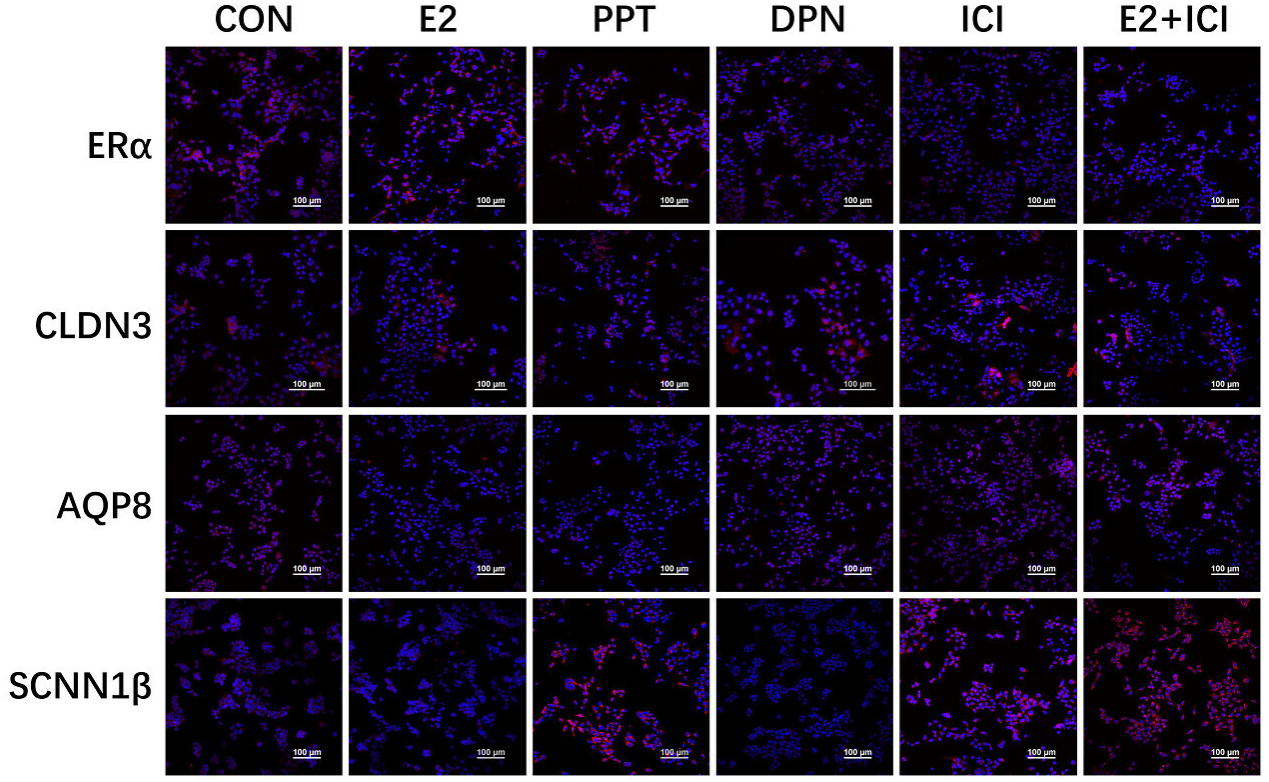
Localization of ERα, CLDN3, AQP8, and SCNN1β in RL95-2 cell line. CON: control group, E2: treat with 10^-6^M oestrogen, PPT: treat with 10^-6^M ERα agonist, DPN: treat with 10^-6^M ERβ agonist, ICI: treat with 10^-6^M oestrogen receptor antagonist, E2+ICI: treat with both 10^-6^M E2 and 10^-6^M ICI. The blue colour: nucleus stained by DAPI. The red colour: localization of target proteins.

The results showed that ERα was localized in the luminal and glandular epithelia of the mouse endometrium (**Figure 4A**). The protein expression of ERα increased by 50% in the 10 IU group compared to the CON group (**Figure 4B**), and ERβ was not obviously expressed (**Figure 4A**). In RL95-2 cell line, ERα localized in cytoplasm. High oestrogen levels and oestrogen receptor agonists increased the expression of ERα, while oestrogen receptor inhibitors can inhibited the expression of ERα (**Figure 5**). This result suggeststhat hyperoestrogenism regulates embryo implantation through ERα.

### 3.9 Localization and western blot analyses of the CLDN3 and OCLN proteins in the mouse uterus during the peri-implantation period

**Figure 6:**
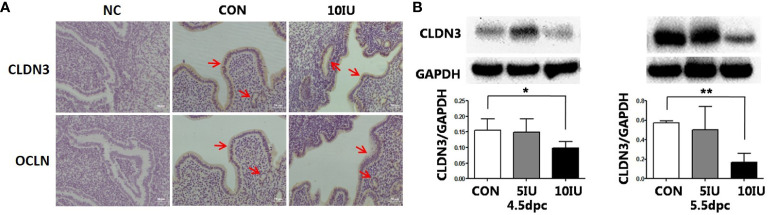
Localization and protein expression of the tight junction proteins CLDN3 and OCLN in the mouse endometrium at 4.5 dpc and 5.5 dpc among the CON, 5 IU and 10 IU groups. **(A)** Localization of CLDN3 and OCLN in the endometria of mice in the CON and 10 IU groups at 5.5 dpc. Bar=50 mm. **(B)** Protein expression of CLDN3 in the endometria of mice in the CON, 5 IU and 10 IU groups at 4.5 dpc and 5.5 dpc. NC: negative control Compared with the CON group, *p<0.05, **p<0.01. The arrows indicate the localization of target protein.

**Figure 7:**
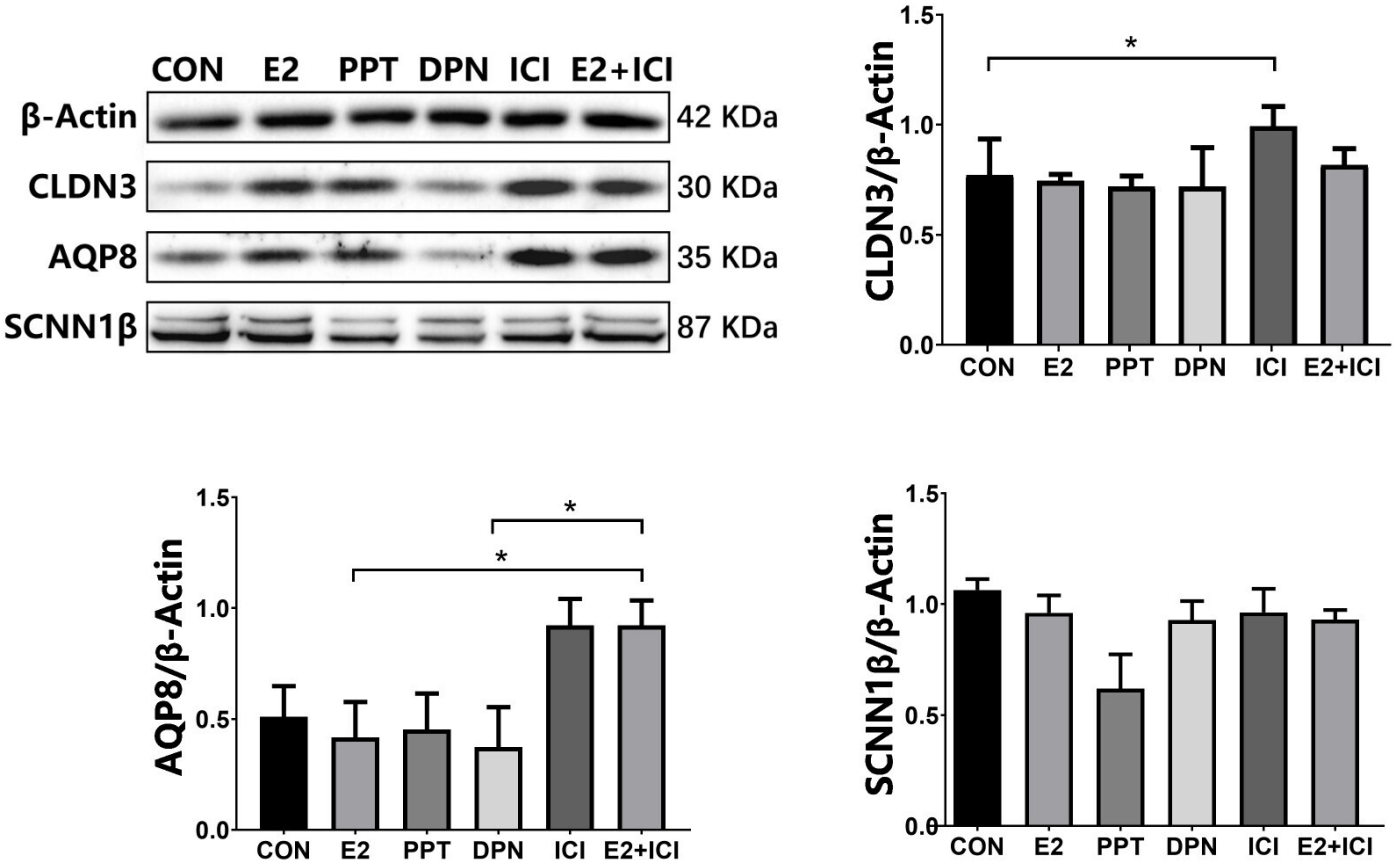
protein expression of CLDN3, AQP8, and SCNN1β in RL95-2 cell line. CON: control group, E2: treat with 10^-6^M oestrogen, PPT: treat with 10^-6^M ERα agonist, DPN: treat with 10^-6^M ERβ agonist, ICI: treat with 10^-6^M oestrogen receptor antagonist, E2+ICI: treat with both 10^-6^M E2 and 10^-6^M ICI. *p<0.05.

At 5.5 dpc, CLDN3 and OCLN were localized in the epithelium of the mouse endometrial cavity (**Figure 6A**). At 4.5 and 5.5 dpc, the CLDN3 protein level in the 10 IU group was lower than that in the CON group (p<0.05 and p<0.01, respectively, **Figure 6B**). In RL95-2 cell line, CLDN3 was mainly localized in membrane. High oestrogen levels and oestrogen receptor agonists depressed the expression of CLDN3, while oestrogen receptor inhibitors can promote the expression of CLDN3 (**Figures 5**, **7**). This result suggeststhat high oestrogen levels can reduce the expression of CLDN3 through ERα.

### 3.10 Localization and western blot analyses of the AQP3, AQP4 and AQP8 proteins in the mouse uterus during the peri-implantation period

**Figure 8:**
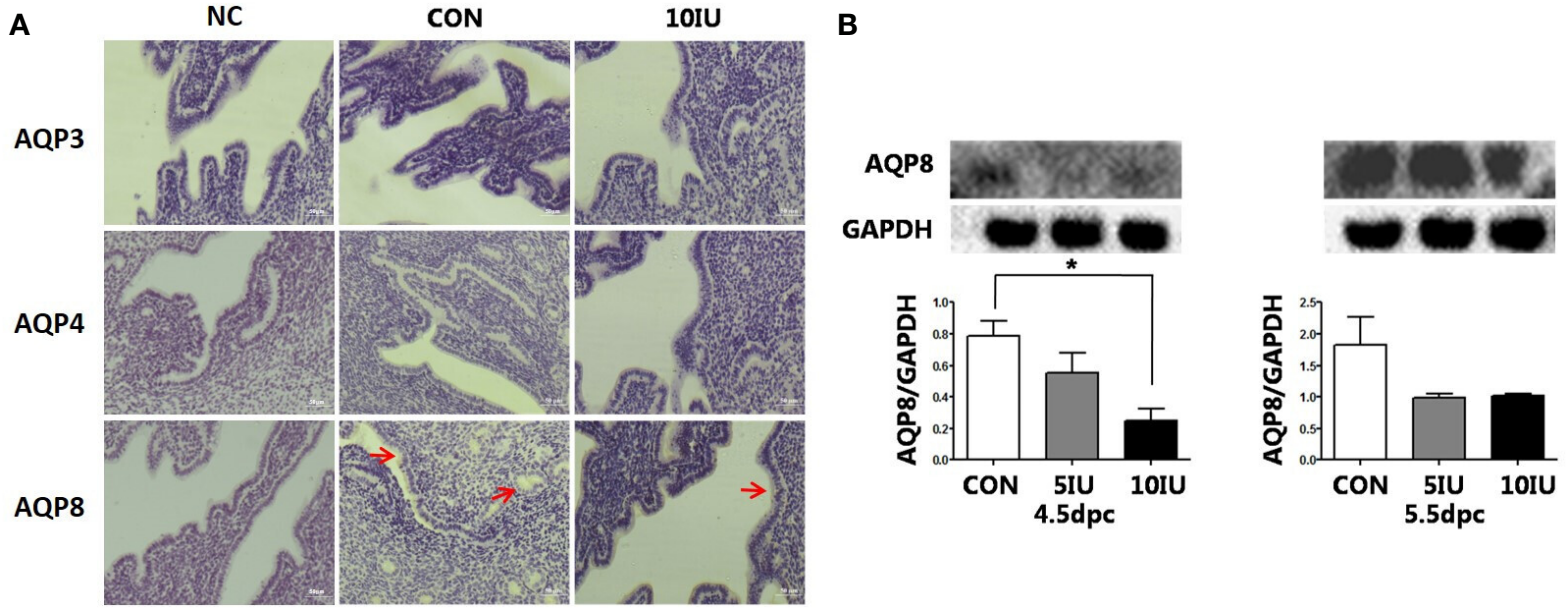
Localization and protein expression of the aquaporins AQP3, AQP4 and AQP8 in the mouse endometrium at 4.5 dpc and 5.5 dpc among the CON, 5 IU and 10 IU groups. **(A)** Localization of AQP3, AQP4 and AQP8 in the endometria of mice in the CON and 10 IU groups at 5.5 dpc. Bar=50 μm. **(B)** Protein expression of AQP8 in the endometria of mice in the CON, 5 IU and 10 IU groups at 4.5 dpc and 5.5 dpc. NC: negative control. Compared with the CON group, *p<0.05. The arrows indicate the localization of target protein.

At 5.5 dpc, AQP3, AQP4, and AQP8 were not obviously expressed in the murine endometrial cavity epithelium or glandular epithelium (**Figure 8A**). At 4.5 dpc, the expression of AQP8 in the 10 IU group was significantly lower than that in the CON group (p<0.05). At 5.5 dpc, the expression of AQP8 in the 10 IU group was lower than that in the CON group (**Figure 8B**). In RL95-2 cell line, AQP8 was mainly localized in cytoplasm. High oestrogen levels and oestrogen receptor agonists depressed the expression of AQP8, while oestrogen receptor inhibitors can promote the expression of AQP8 (**Figures 5**, **7**). This result suggeststhat high oestrogen levels can also reduce the expression of aquaporin AQP8 through ERα.

### 3.11 Localization and western blot analyses of the SCNN1α, SCNN1β and SCNN1γ proteins in the mouse uterus during the peri-implantation period

**Figure 9:**
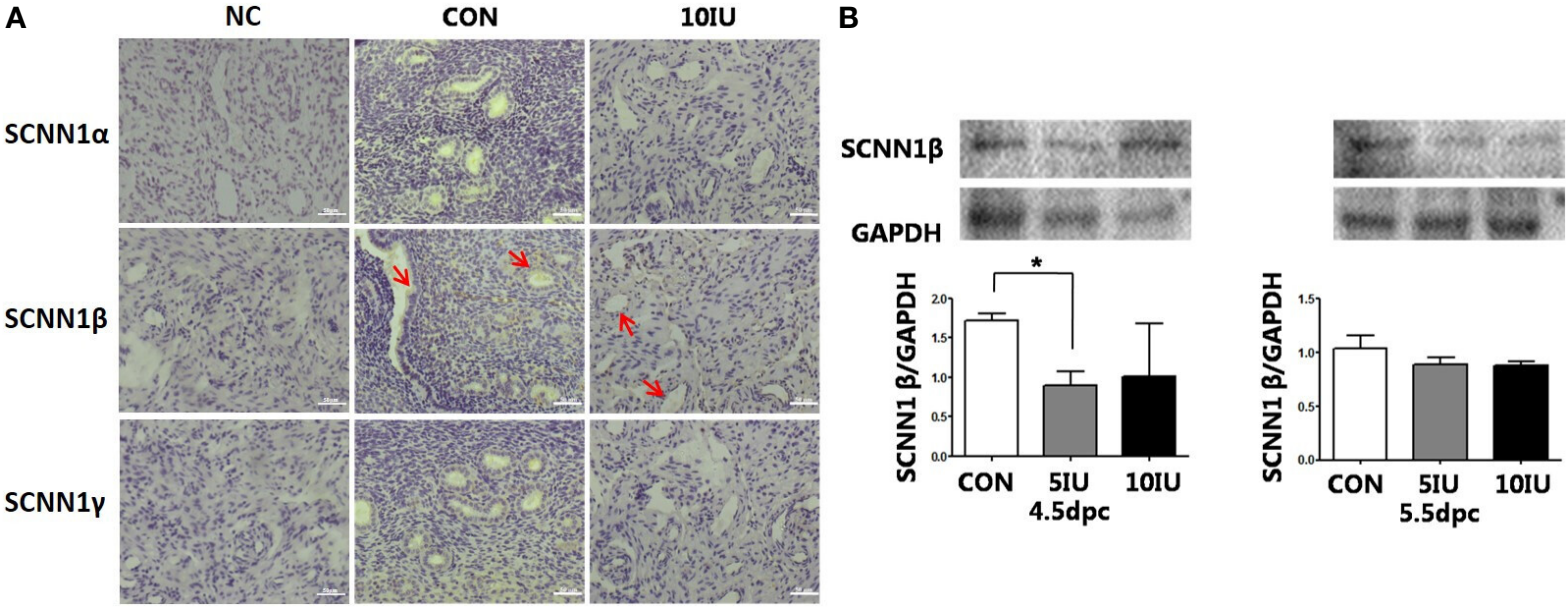
Localization and protein expression of the sodium channel proteins SCNN1α, SCNN1β and SCNN1γ in the mouse endometrium at 4.5 dpc and 5.5 dpc among the CON, 5 IU and 10 IU groups. **(A)** Localization of SCNN1α, SCNN1β and SCNN1γ in the endometria of mice in the CON and 10 IU groups at 5.5 dpc. Bar=50 μm. **(B)** Protein expression of SCNN1β in the endometria of mice in the CON, 5 IU and 10 IU groups at 4.5 dpc and 5.5 dpc. NC: negative control. Compared with the CON group, *p<0.05. The arrows indicate the localization of target protein.

At 5.5 dpc, SCNN1α and SCNN1γ were not obviously expressed. SCNN1β was localized in the murine endometrial cavity epithelium and glandular epithelium (**Figure 9A**).

At 4.5 dpc, the expression of SCNN1β in the 5 IU group was significantly lower than that in the CON group (p<0.05); its expression in the 10 IU group was reduced, but the difference was not significant. At 5.5 dpc, the expression of SCNN1β did not significantly differ among the three groups (**Figure 9B**). In RL95-2 cell line, SCNN1β was mainly localized in cytoplasm and showed polarity. High oestrogen levels and oestrogen receptor agonists depressed the expression of SCNN1β, while oestrogen receptor inhibitors can promote the expression of SCNN1β (**Figures 5**, **7**).

## 4 Discussion

Exogenous gonadotropins are used to induce superovulation in humans and animals to increase the number of oocytes and embryos, thereby increasing the success rate of pregnancy (34). The PMSG/HCG regimen has been used in many laboratories to induce superovulation in mice for more than 60 years (35). However, superovulation in female mice increases the number of unhealthy follicles that are ovulated and leads to an increase in the number of low-quality oocytes (36–38). Superovulation treatment can induce changes in the maternal fallopian tubes that make them unsuitable for the transport of oocytes and cause alterations in the uterine environment, thereby impairing implantation and preventing subsequent pregnancy in female mice (39). Therefore, gonadotropin superovulation therapy seems to have an adverse effect on the maternal environment.

The doses of PMSG and hCG that are commonly used to construct mouse models of superovulation are 5 IU (40), 7.5 IU (41, 42), and 10 IU (43). The 10 IU dose was previously verified to seriously decrease the embryo implantation rate (43), but the specific mechanism has not been studied. This study revealed that the administration of PMSG and HCG to stimulate ovulation in mice simulated the increase in the ovarian volume caused by the clinical ovulation stimulation cycle, induced a continuous increase in serum oestrogen levels, and induced abnormal or failed embryo implantation in a dose-dependent manner.

E2 is reportedly a key determinant of the duration of the endometrial receptive implantation window (9) and mainly acts through nuclear oestrogen receptors (mainly ERα but not ERβ) (44, 45). Recently, Chai et al. compared the effects of high serum E2 levels on endometrial steroid receptor levels during the gonadotropin stimulation cycle and the natural cycle. Their results showed that oestrogen receptor expression was significantly reduced during the stimulation cycle (46). The results of this study showed that ERα expression but not ERβ expression was increased by 50% in the 10 IU group compared to the CON group at 5.5 dpc. However, the difference is not statistically significant. This finding confirms that at superphysiological concentrations, E2 exerts its effects *via* ERα.

Secreted uterine cavity fluid initially allows the transport of and provides support for sperm and untransferred embryos, while the absorption of uterine cavity fluid in early pregnancy leads to the closure of the cavity and allows the blastocyst to establish close contact with the uterine epithelium. The content and volume of fluid in the uterine cavity are jointly regulated by TJs, water channels, and ion channels, among others (47).

The results of this study show that an imbalance in uterine cavity fluid during embryo implantation may cause embryo implantation failure. Embryo implantation was delayed after ovulation induction, the spacing between embryos was uneven, the number of embryos was reduced, and the incidence of abnormal implantation and miscarriage was high.

There is some evidence that TJs play a role in the uterus and implantation. Previous studies have shown that both CLDN3 and CLDN7 are expressed in the luminal and glandular epithelium in the mouse endometrium during the oestrous cycle and that CLDN10 is expressed in only the glandular epithelium. At 4.5 dpc, the time point at which embryo implantation occurs, the CLDN3 protein is localized at the top of the epithelium, while CLDN7 is not expressed in the epithelium at the implantation site. Moreover, CLDN3 and CLDN7 are not expressed in the matrix, but CLDN10 is strongly expressed in the primary decidual area (48). CLDN3 mRNA and protein are highly expressed in the luminal epithelia of mice on the 3rd and 4th days of pregnancy, but by the 5th day of pregnancy, when blastocysts are implanted, the expression of CLDN3 is downregulated. At this time, the downregulation of CLDN-3 expression may be beneficial for the loss of the luminal epithelial barrier, and changes in the expression and morphology of TJ proteins help blastocysts to invade the endometrium (49–52). At the same time, CLDN3 and CLDN10 are expressed in human endometrial epithelial cells (48). Thus, CLDN3 plays an important role in the process of embryo implantation. In this study, we evaluated the dynamic expression of CLDN3 and OCLN in mice subjected to PMSG+HCG-induced ovulation induction during the peri-implantation period. The results showed that CLDN3 and OCLN were localized in the luminal epithelium of the mouse endometrium at 5.5, 4.5 and 5.5 dpc and that the expression of CLDN3 in the 10 IU group was lower than that in the CON group. The decreased expression of CLDN3 is mainly mediated by ER α, which can be blocked by oestrogen receptor blocker ICI182780. These results suggest that the reduced CLDN3 expression in the present study suggest that the embryo implantation in the 10 IU group was affected by the highly female bodily environment.

The expression of AQP3 in the middle and late stages of human endometrial secretion is significantly higher than that in other stages. The protein expression of AQP5 in uterine cavity epithelial cells was shown to be increased in pregnant rats subjected to controlled ovarian hyperstimulation (COH) compared to normal pregnant rats. At the time of implantation, the distribution of AQP5 staining in rats subjected to COH was altered (53). There was no significant difference in the pregnancy rate between AQP8 knockout mice and wild-type mice. Compared with that in wild-type control mice, the number of embryos in pregnant AQP8 knockout mice was shown to be significantly increased (54).

Ovarian stimulation alters the expression of D4 Aqp3, Aqp5 and Aqp8 (33). Our results suggest that AQP8 plays a role in ovulation induction-induced hyperoestrogensim leading to abnormal or failed embryo implantation. Hyperoestrogenism decreased the expression of AQP8 through ERα. The decreased expression of AQP8 may affect the embryo implantation process by regulating the uterine fluid.

It was previously demonstrated that ENaC-α in the mouse endometrium is activated to the greatest extent at the time of implantation. ENaC deficiency or low expression of ENaC in the endometrium may lead to a low pregnancy success rate or abortion in patients undergoing *in vitro* fertilization (IVF) (55). In this study, high oestrogen levels during ART led to reduced expression levels of SCNN1β through ERα, which may account for the reduced endometrial receptivity, abnormal embryo implantation or implantation failure.

At physiological concentrations, E2 and P regulate the volume of fluid in the uterine cavity during embryo implantation. In this study, we found that superphysiological concentrations of E2 significantly affected the expression of TJ, water channel, and sodium channel proteins in endometrial epithelial cells *in vivo*. This result provides a possible mechanism for low endometrial receptivity during the COH cycle. We found that superphysiological concentrations of E2 played a role in destroying TJs, water channels, and sodium channels. At superphysiological concentrations, E2 may impair embryo implantation by inducing fluid imbalance in the peri-implantation uterine cavity through ERα, thus hindering endometrial receptivity for implantation. We have shown that uterine fluid imbalance during COH is mediated by abnormal downregulation of CLDN3, AQP8 and SCNN1β expression. Our research lays the foundation for further research on the role of these factors in clinical phenomena such as embryo implantation failure and miscarriage.

## 5 Conclusions

These results suggest that in the 10 IU group, the implantation of mouse embryos was affected by the highly female bodily environment, implantation was delayed, the embryos were unevenly spaced, and the abortion rate was high, while the oestrogen environment in the 5 IU group had a smaller effect on receptivity. High oestrogen levels during ART alter the expression levels of TJ, aquaporin and sodium channels proteins through ERα, thereby destroying the TJs, water and sodium channels between the epithelium of the endometrial cavity and resulting in reduced endometrial receptivity, delayed implantation, and abnormal embryo implantation. These factors are associated with E2 and are the result of the co-regulation of multiple E2-related pathways.

## 6 Limitations

Further research is needed to clarify the downstream mechanism *via* which hyperoestrogensim related to ART mediates the regulation of TJ, aquaporin and sodium channel proteins.

## Data availability statement

The raw data supporting the conclusions of this article will be made available by the authors, without undue reservation.

## Ethics statement

The animal study was reviewed and approved by the Experimental Animal Ethics Committee of the University of Nanjing Medical University.

## Author contributions

XX wrote the draft of the manuscript. YZ performed the data analyses. MC, XY and LG conducted animal experiments and acquired the data. LQ, WW and YC reviewed and edited the manuscript. JL designed the study. All authors contributed to the article and approved the submitted version.
