# Supplementary material for: Adverse effect of assisted reproductive technology-related hyperoestrogensim on the secretion and absorption of uterine fluid in superovulating mice during the peri-implantation period
Source: Front Endocrinol (Lausanne). 2023 Mar 6;14:859204. doi: 10.3389/fendo.2023.859204 (PMC10027003; doi:10.3389/fendo.2023.859204)
Supplement: Supplementary file 1 [file DataSheet_1.docx]

 **Supplemental Fig. 1 Weights of the mice in the CON, 5 IU and 10 IU groups**

P/NaCl: day of the intraperitoneal injection of PMSG/normal saline; H/NaCl: day of the intraperitoneal injection of HCG/normal saline; 1.5 dpc, 3.5 dpc and 5.5 dpc: the day of the suppository was recorded as 0.5 dpc, the second day was recorded as 1.5 dpc, the 4th day was recorded as 3.5 dpc, and the 6th day was recorded as 5.5 dpc.


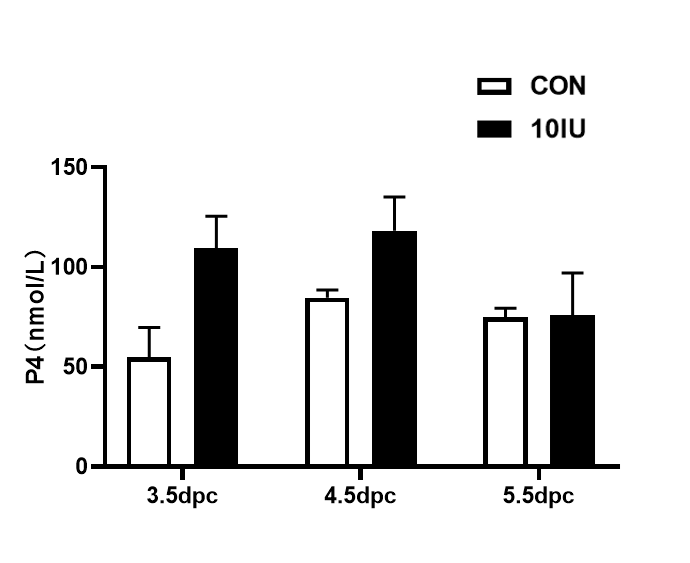


**Supplemental Fig. 2 Serum progesterone levels in the CON and 10 IU groups during the peri-implantation period**

dpc: days post-coitum.

P4: progesterone
